# Sex-specific differences in risk factors and outcomes for long-term mechanical ventilation: a longitudinal cohort analysis of claims data

**DOI:** 10.1038/s41598-025-22399-z

**Published:** 2025-10-08

**Authors:** Franziska C. Trudzinski, Benjamin Neetz, Jana Dahlhoff, Philipp Höger, Axel Kempa, Claus Neurohr, Armin Schneider, Felix J. F. Herth, Biljana Joves, Joachim Szecsenyi, Elena Biehler, Thomas Fleischhauer, Janina Schubert-Haack, Thomas Grobe, Timm Frerk, The PRiVENT-Study Group, Franziska C. Trudzinski, Benjamin Neetz, Jana Dahlhoff, Axel Kempa, Claus Neurohr, Armin Schneider, Felix J. F. Herth, Biljana Joves, Joachim Szecsenyi, Elena Biehler, Janina Schubert-Haack, Thomas Grobe, Timm Frerk, Gabriele Iberl, Julia Dorothea Michels-Zetsche, Michael Müller, Andreas Rheinhold, Ahmed Ehab, Alessandro Ghiani, Nina Lutz, Swenja Walcher, Konstantinos Tsitouras, Joanna Paderewska, Selina Briese, Christoph Rauch, Patrick Gehrig, Joachim Sugg, Susanne Hirschmann, Thomas Fleischhhauer, Gerhard Fuchs, Nicola Litke, Markus Qreini, Michel Wensing, Anja Klingenberg, Teresa Byczkowski, Manuel Feißt, Simone Britsch, Christa Straub, Claude Jabbour, Michael Hahn, Jörg Krebs, Peter-Tobias Graf, Petra Denzer, Uta Merle, Monica Boxberger, Mascha Fiedler-Kalenka, Guido Hundt, Jens Regula, Thushira Weerawarna, Miriane Bomeken, Lisa Amega, Shumallah Basit, Sebastian Stier, Matthias Körner, Jens Müller, Sergej Markin, Ute Oltmanns, Oliver Gorgs, Mark Hackbarth, Sebastian Münz, Dominik Scharpf, Thomas J. Dengler, Mathias Borst, Brigitte Mayer, Wolfgang Reikow, Markus Kredel, Patrick Keppeler, Konstantin Frey, Holger Wolff, Florian Seidlitz, Stefanie Bientzle, Boris Nohé, Sebastian Allgäuer, Alexej Schöpp, Jörg Winckelmann, Christoph Schlegel, Imke Hübner, Andrzej Kuzniar, Helene Häberle, Reimer Riessen, Benjamin Schempf, Ingo Rebenschütz, Andreas Straub, Marc Kollum, Markus Winter, Paul Hartveg, Andreas Junginger, Thomas Abt, Mathias Vogel, Ralf Völker, Thomas Wiesmann

**Affiliations:** 1 Department of Pneumology and Critical Care, Thoraxklinik Heidelberg gGmbH, Translational Lung Research Center Heidelberg (TLRC-H), Member of the German Center for Lung Research (DZL), Heidelberg, Germany; 2 Department of Pneumology and Critical Care, SLK-Klinik Löwenstein, Löwenstein, Germany; 3 Department of Pneumology and Respiratory Medicine, RBK Lungenzentrum Stuttgart at Robert-Bosch-Krankenhaus, Stuttgart, Germany; 4 Department of Anaesthesiology and Intensive Care Medicine Waldburg-Zeil Kliniken, Wangen Im Allgäu, Germany; 5 Department of General Practice and Health Services Research, University Hospital Heidelberg, Heidelberg, Germany; 6 aQua Institute for Applied Quality Improvement and Research in Health Care, Göttingen, Germany; 7 Institute of Medical Biometry, University of Heidelberg, Heidelberg, Germany; 8 Department of Cardiology, Angiology, Haemostaseology and Medical Intensive Care, University Medical Center Mannheim, Mannheim, Germany; 9 European Center for Angioscience (ECAS) and German Center for Cardiovascular Research (DZHK), Partner Site Heidelberg/Mannheim, Mannheim, Germany; 10 Department of Anaesthesiology and Medical Intensive Care, University Medical Center Mannheim, Mannheim, Germany; 11 Department of Gastroenterology, Hepatology and Infectious Diseases, University Hospital Heidelberg, Heidelberg, Germany; 12 Department of Anaesthesiology, University Hospital Heidelberg, Medical Faculty, University of Heidelberg, Heidelberg, Germany; 13 Department of Neurology, SRH Kurpfalzkrankenhaus, Heidelberg, Germany; 14 Department of Pneumology, Respiratory and Sleep Medicine, Siloah St. Trudpert Klinikum Pforzheim, Pforzheim, Germany; 15 Department of Pneumology, Städtisches Klinikum Karlsruhe, Karlsruhe, Germany; 16 Department of Anaesthesiology and Intensive Care Medicine, GRN-Klinik Schwetzingen, Schwetzingen, Germany; 17 Department of Pneumology, Helios Klinikum Pforzheim, Pforzheim, Germany; 18 Department of Anaesthesiology, Emergency Medicine and Intensive Care Medicine, St. Josefskrankenhaus, Heidelberg, Germany; 19 Department of Cardiology, Angiology, Pneumology and Internistic Intensive Care Medicine, SLK Klinikum am Gesundbrunnen, Heilbronn, Germany; 20 Department of Cardiology, Angiology and Intensive Care Medicine, SLK Klinikum am Plattenwald, Bad Friedrichshall, Germany; 21 Department of Anaesthesiology and Intensive Care Medicine, Caritas-Krankenhaus Bad Mergentheim, Bad Mergentheim, Germany; 22 Clinic for Internal Medicine (Medizinische Klinik II), Klinikum Heidenheim, Heidenheim, Germany; 23 Department of Anaesthesiology and Intensive Care Medicine, Klinikum Crailsheim, Crailsheim, Germany; 24 Department of Anaesthesiology, Intensive Care-, Emergency- and Pain Medicine, Ostalb Klinikum Aalen, Aalen, Germany; 25 Department of Anaesthesiology, Intensive Care and Pain Medicine, St. Anna-Virngrund-Klinik Ellwangen, Kliniken Ostalb, Ellwangen, Germany; 26 Department of Anaesthesiology, Surgical Intensive Care- and Emergency Medicine, Hohenloher Krankenhaus, Öhringen, Germany; 27 Department of Intensive Care Medicine, Die Filderklinik, Filderstadt-Bonlanden, Germany; 28 Department of Anaesthesiology and Intensive Care Therapy, SRH Klinikum Karlsbad-Langensteinbach, Karlsbad, Germany; 29 Center for Anaesthesiology, Intensive Care Medicine, Emergency Medicine and Pain Medicine, Zollernalb Klinikum, Albstadt/Balingen, Germany; 30 Department of Anaesthesiology and Surgical Intensive Care Medicine, Robert-Bosch-Krankenhaus, Stuttgart, Germany; 31 Department of Cardiology and Angiology, Robert-Bosch-Krankenhaus, Stuttgart, Germany; 32 Department of Anaesthesiology and Intensive Care Medicine, Diakonie-Klinikum Stuttgart, Stuttgart, Germany; 33 Department of Anaesthesiology, University- and Rehabilitation Clinic RKU Ulm, Ulm, Germany; 34 Department of Cardiology, Angiology and Intensive Care Medicine, Krankenhaus Landkreis Freudenstadt, Freudenstadt, Germany; 35 Department of Anaesthesiology and Intensive Care Medicine, University Hospital Tübingen, Tübingen, Germany; 36 Department of Internistic Intensive Care Medicine, University Hospital Tübingen, Tübingen, Germany; 37 Medical Clinic II, Klinikum Am Steinenberg, Kreiskliniken Reutlingen, Reutlingen, Germany; 38 Department of Anaesthesiology and Intensive Care Medicine, Klinikum Landkreis Tuttlingen, Tuttlingen, Germany; 39 Department of Anaesthesiology, Intensive Care Medicine and Pain Medicine, St. Elisabethen Klinikum Ravensburg, Ravensburg, Germany; 40 Department of Cardiology and Internistic Intensive Care Medicine (1. Medizinische Klinik), Hegau-Bodensee-Klinikum Singen, Singen, Germany; 41 Department of Internistic Intensive Care Medicine, Alb-Donau Klinikum Blaubeuren, Blaubeuren, Germany; 42 Department of Internal Medicine and Cardiology, Alb-Donau Klinikum Ehingen, Ehingen, Germany; 43 Department of Anaesthesiology, Intensive Care Medicine, Emergency Medicine and Pain Medicine, Oberschwabenklinik Westallgäu-Klinikum, Wangen, Germany; 44 Department of Cardiology, Anaesthesiology, Pneumology and Interdisciplinary Intensive Care Medicine, Medizin Campus Bodensee Klinikum Friedrichshafen, Friedrichshafen, Germany; 45 Department of Internistic Intensive Care Medicine and Emergency Medicine, Diak Klinikum Diakoneo, Schwäbisch Hall, Germany; 46 Department of Anaesthesiology, Surgical Intensive Care Medicine and Pain Therapy, Diak Klinikum Diakoneo, Schwäbisch Hall, Germany

**Keywords:** Long-term invasive mechanical ventilation, Invasive home mechanical ventilation, Weaning, Weaning failure, Prolonged weaning, Prognostic factors, Predictive model, Medical research, Biomarkers

## Abstract

Sex differences in intensive care medicine, particularly regarding risk factors for (MV), are underexplored. This study aimed to investigate sex-related risk factors for long-term MV. We analyzed claims data from patients aged ≥ 30, with at least one comorbidity, who received MV (≥ 96 h) between 2015 and 2018. Data covered 365 days prior to hospitalization and 30 days post-discharge. Sex differences in 29 predefined risk factors were assessed. Over 12,000 hospitalizations were analyzed, with 37.8% of patients being female. Women were older (71.3 ± 11.6 vs. 69.9 ± 11.2 years, p < 0.001) and more often transferred from nursing homes (4.5% vs. 2.7%, p < 0.001). Men required invasive long-term ventilation more often (33.8% vs. 31.2%, p = 0.004) and had more ventilation hours (400.0 ± 377.9 vs. 373.0 ± 341.8, p < 0.001). Among pre-existing conditions, women had more thyroiditis and rheumatic mitral valve disease, while men had more COPD, cardiac arrhythmia, eating disorders, and acute pancreatitis. Men were more likely to undergo bronchoscopy, autologous blood transfusion, or Extracorporeal life support (ECLS) within 96 h of MV. Multivariate analyses revealed stronger associations between certain risk factors and long-term MV by sex. For men, thyroiditis (OR 2.7 vs. 1.4 in women), cerebral infarction (OR 2.3 vs. 1.4), and acute pancreatitis (OR 3.2 vs. 1.0) were more strongly linked to weaning failure. For women, cerebrospinal fluid surgery (OR 5.7 vs. 2.0 in men) and acute pancreatitis (OR 4.7 vs. 3.0 in men) had stronger associations with long-term MV. ECLS and eating disorders were only linked to long-term MV in men. This study highlights sex differences in risk factors and outcomes for long-term MV, suggesting the need for sex-specific management strategies.

**Trial registration:** The PRiVENT study was retrospectively registered at ClinicalTrials.gov (NCT05260853). Registered at March 2, 2022.

**Supplementary Information:**

The online version contains supplementary material available at 10.1038/s41598-025-22399-z.

## Introduction

Patient sex has long played a subordinate role in critical care medicine, despite well-documented sex differences in the physiology, burden of comorbidities and therapeutic responses of critically ill patients ^1^. However, with an increasing focus on individualized treatment approaches, this factor is coming to the fore. Weaning is a critical step in the management of mechanically ventilated patients. The timing of extubation requires careful consideration, as both premature and delayed extubation can cause harm^2,3^. This is particularly relevant in patients at risk for long-term mechanical ventilation, where optimizing extubation timing may influence outcomes and reduce complications. Premature extubation risks respiratory failure and the need for reintubation, which can lead to complications and poorer outcomes. Conversely, delayed extubation prolongs mechanical ventilation unnecessarily, increasing the risk of ventilator-associated pneumonia, muscle weakness and psychological distress. Approximately 40% of intubated patients in the ICU are women ^4–6^. In a post-hoc analysis of a large clinical trial, Thille et al. investigated sex differences in the risk of extubation failure in ICU patients at high risk of extubation failure ^7^. They found that men had a significantly higher rate of reintubation within 48 h, with male sex independently associated with an increased risk of reintubation within 7 days ^7^. Conversely, women are more likely to develop laryngeal oedema after extubation ^8^. This is thought to be due to the relatively larger size of the tracheal tube in relation to the larynx and trachea in women, which may cause greater mechanical trauma and subsequent swelling ^9^. Röser et al. recently conducted a monocentric retrospective study of patients undergoing prolonged weaning. They showed that in this specific patient population, sex itself was not a risk factor for weaning failure, defined as the combined endpoint of long-term mechanical ventilation and mortality in the weaning center. However, the risk factors identified for weaning failure differed between men and women, highlighting the importance of considering sex differences in this context ^10^. A comprehensive study investigating sex differences in the risk factors for weaning failure or the subsequent need for long-term mechanical ventilation in the acute intensive care setting has yet to be conducted. The aim of our study was to address this gap by examining a large cohort of patients at risk for long-term mechanical ventilation. To achieve this, we analyzed claims data from patients over 30 years of age who were ventilated for ≥ 96 h and had at least one internal comorbidity. The data were provided by the largest statutory health insurance company in Germany. This study is a sub-project of the multi-center PRiVENT study ^11^, which aims to develop strategies to prevent long-term IMV and improve expertise through knowledge acquisition, training ^12^ and networking ^11,13^.

## Methods

The present study builds on preliminary work that has already been published ^14,15^ and adheres to the STROBE (Strengthening the Reporting of Observational Studies in Epidemiology) guidelines. The analysis used claims data from Allgemeine Ortskrankenkasse Baden-Württemberg (AOK-BW), one of the largest health insurers in Germany, covering approximately 4.38 million insured people, or about 5.96% of the country’s statutory health insurance population. This represents around 43% of the population of the state of Baden-Württemberg, whose population is comparable to that of Belgium and larger than Denmark or Norway. The data were provided in pseudonymized form and analyzed by the Institute for Applied Quality Improvement and Research in Health Care (aQua) in close collaboration with a team of experienced clinicians. The PRiVENT study was registered at ClinicalTrials.gov (NCT05260853). Registered at March 2, 2022.

### Ethics declarations

The study protocol (version 1.0) was approved by the Ethics Committee of the Medical Faculty of Heidelberg University (S-352/2018) prior to the study’s initiation on 18th September 2020. An amended version of the protocol (version 1.4) received approval on 27th April 2021. All methods were carried out in accordance with relevant guidelines and regulations. Data protection was ensured in compliance with the European General Data Protection Regulation (GDPR), the Baden-Württemberg State Data Protection Act, and the Federal Data Protection Act. The data protection concept for this study was reviewed and approved by the data protection officer of Heidelberg University Hospital. The need for informed consent was waived by the ethics committee of the Ethics Committee of the Medical Faculty of Heidelberg University due to the retrospective nature of the study.

### Patients

The study included AOK Baden-Württemberg insured patients who underwent invasive mechanical ventilation for ≥ 96 h, were over 30 years old, and had a medical comorbidity, [One of the following International Statistical Classification of Diseases and Related Health Problems (ICD-10) codes, coded in the 365 days prior to the ventilation case: J44, M41, J60-J70, J84, I50, I25, E10-E14, E66.01, E66.02, C00-C97, F05, F10.4-16.4 (in each case those ending in 0.4), F18.4, F19.4, F20-29, G62.80, G72.80, N17, N18] with hospital admissions between January 2015 and November 2018. Patients with previous invasive home MV or neuromuscular disease unsuitable for ventilator weaning were excluded. To capture pre-existing conditions and assess the sustainability of weaning, patients needed to be insured with AOK Baden-Württemberg for the 365 days before and 30 days after discharge. The methodology has been published elsewhere ^14^. In this study, the observation period was extended, and patients who died within the first 11 days, previously excluded in the prior study, were included in the current analysis.

### Analysis of claims data

The analysis is based on routine health insurance data, utilizing ICD-10 codes and official German classification of operational procedures (OPS) codes to identify hospital and patient characteristics associated with an increased risk of long-term invasive ventilation as well as detailed data on master data, data on aids and care. The methodology, including detailed analyses, has been previously published. In the original analysis, 3-digit ICD codes (and selected 4-digit codes) and 4-digit OPS codes (and selected 5-digit codes) were examined across different time periods. Frequencies of diagnoses and procedures observed in patients requiring long-term ventilation were compared with expected frequencies in the general population. Significant differences underwent further investigation. Additional data included prescriptions for medical aids, procedures conducted in the first 96 h of intensive care—particularly the first 24 h relevant for intubation and related OPS codes—and data from the year prior to admission and the 30 days post-discharge. The day of intubation was defined as day 0. Predictors were identified stepwise in collaboration with a consulting team, and their independent effects were assessed using logistic regression models ^14^. In the current analysis, these previously identified risk factors were re-evaluated to specifically examine their interaction with sex and the influence of sex on long-term ventilation outcomes. Supplementary Fig. 1 provides an overview of the time periods for predictor collection and result assessment.

### Outcomes

Long-term invasive mechanical ventilation (IMV) was defined as follows: evidence of invasive home mechanical ventilation after discharge, a total ventilation duration of ≥ 500 h, or re-hospitalization with (re)prolonged ventilation (IMV ≥ 96 h). The criteria and operational definitions of outcomes are listed in supplementary Table  1.

### Statistical methods

Binary logistic regression models were estimated separately by sex to predict the risk of long-term IMV. The selection of predictors was based on previous work in which sex was only one predictor; however, no sub-analyses were conducted to identify sex-specific risk factors. To work out sex differences, all predictors were left in the model, even if they did not prove to be significant for one sex. The statistical analyses were performed with SAS Enterprise Guide 7.1; figures were created with R 4.4.1 using RStudio 2024.04.02.

## Results

### Hospitalizations

**Fig. 1:**
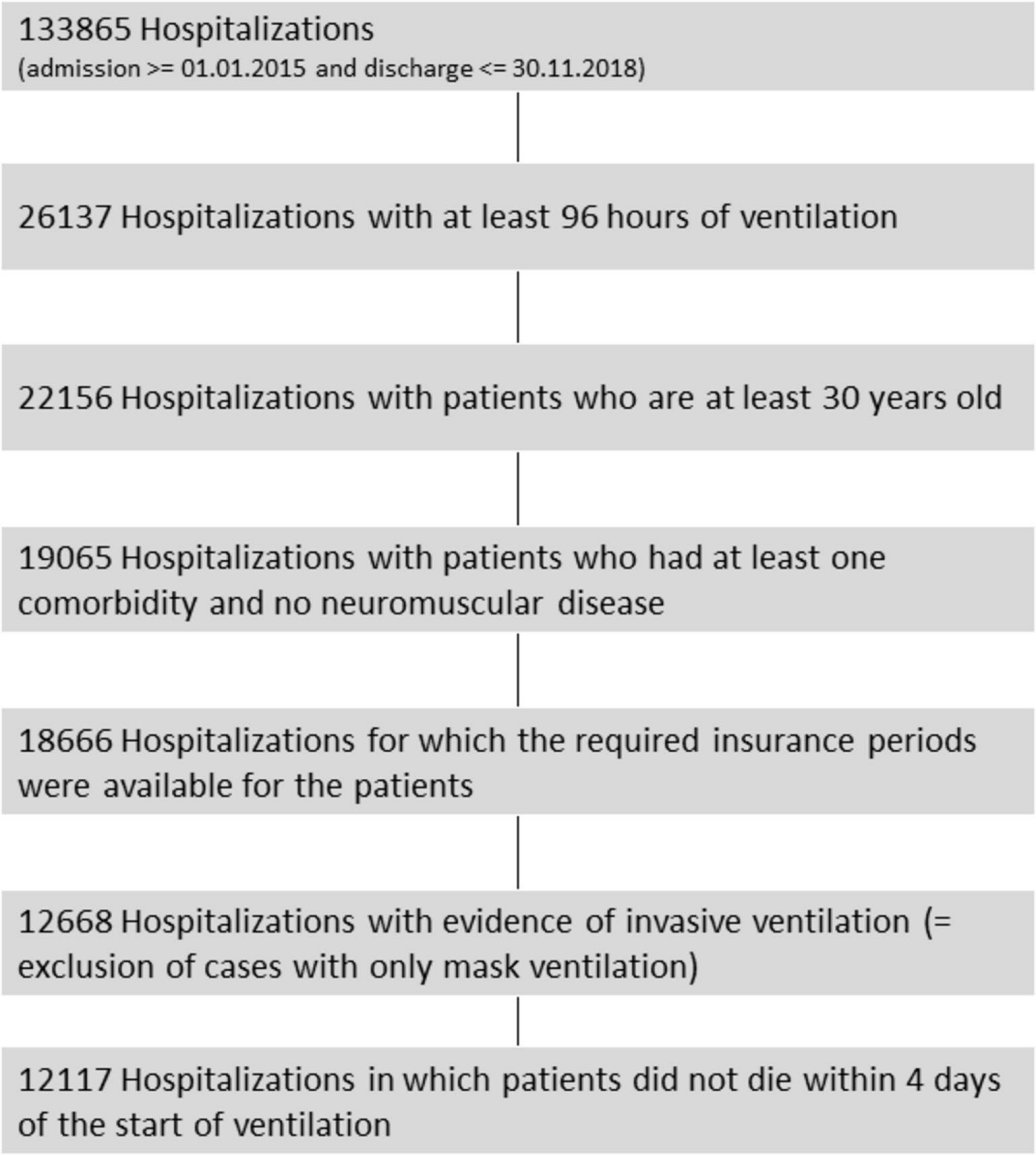
Consort diagram of inclusions and exclusions.

A total of 12,117 hospitalizations involving 11,263 patients who received invasive mechanical ventilation for at least 96 h were included in the analysis. Among the patients, 37.9% were female. Figure 1 shows a flowchart of the analyzed hospitalizations. Female patients were slightly older, with a mean age of 71.3 ± 11.6 years, compared to 69.9 ± 11.2 years for male patients (p < 0.001). Men were more likely to require invasive long-term ventilation, with 33.8% of men requiring this treatment compared to 31.2% of women (p = 0.004). Hospital mortality rates were similar for both sexes, with 37.0% of women and 37.3% of men dying in hospital (p = 0.701). Regarding mechanical ventilation hours, men had a higher mean number of mechanical ventilation hours (400.0 ± 377.9) compared to women (373.0 ± 341.8) (p < 0.001). Table 1 provides an overview of the baseline data and key characteristics of the index hospitalization.

**Table 1:** Overview of baseline data and key characteristics of index hospitalisations by sex.

	**All**	**Female**	**Male**	**p**
Hospitalisations	12,117	4588	7529	
Patients	11,263	4261	7002	
Age	70.4 ± 11.4	71.3 ± 11.6	69.9 ± 11.2	< 0.001
Invasive long-term ventilation*	32.8%	31.2%	33.7%	0.004
mortality rate in hospital	37.2%	37.0%	37.3%	0.701
hours of ventilation	389 ± 364.9	373 ± 341.8	400 ± 377.9	< 0.001
Evidence of home invasive ventilation after discharge within 30 days*	4.7%	4.6%	4.8%	0.758
Total duration of ventilation ≥ 500 h	54.2%	53.4%	54.7%	0.183
Re-hospitalisation with (re)prolonged ventilation	13%	12.8%	13.1%	0.583

*This endpoint combines several factors related to home mechanical ventilation (HMV) and patient care. It includes the initiation of home mechanical ventilation, invasive HMV after weaning failure and within 30 days, as well as the control or optimization of previously initiated HMV within the same time frame. Additionally, the termination of previously started home ventilation within 30 days is considered. The prescription of tracheostomy ventilator aids after the start of ventilation and within 30 days of discharge is also a key factor. For inpatient care, long-term dependence on a respirator after the initiation of ventilation is considered, along with the care of a tracheostoma within 30 days after discharge.

### Prevalence of predefined risk factors by sex

Risk factors were categorized into the following groups: baseline predictors, pre-existing conditions (within 365 days prior to the index case), diseases (medical history, admission diagnosis and conditions during the index case), operations and procedures within 365 days prior to the index case and operations and procedures operations and procedures during the index case until the third day after the first documented intubation (max 96 h). The analysis revealed that women were more likely to be transferred from nursing homes than men (4.5% vs. 2.7%; p < 0.001). Among the pre-existing conditions, women were more likely to have thyroiditis (2.3% vs. 0.4%; p < 0.001) and rheumatic mitral valve disease (2.3% vs. 0.6%; p < 0.001). On the other hand, men were more likely to have chronic obstructive pulmonary disease (31.5% vs. 29.3%; p = 0.0118), cardiac arrhythmia (1.2% vs. 0.6%; p = 0.0012), eating disorders (1.1% vs. 0.8%; p = 0.0015), or acute pancreatitis (0.7% vs. 0.3%). During the first 96 h of mechanical ventilation, men were more likely to receive bronchoscopy (36.1% vs. 33.4%; p = 0.0029), autologous blood collection and transfusion (3.0% vs. 2.1%; p = 0.0064), or extracorporeal lung support (3.9% vs. 2.6%; p < 0.001). In multivariate analysis of the predefined set of 29 risk factors, neither age nor sex emerged as significant risk factors for long-term ventilation. Pre-existing medical conditions such as thyroiditis, eating disorders, rheumatic mitral valve disease, and pneumothorax were identified as independent risk factors, while peritonitis and dementia were associated with a beneficial impact on subsequent long-term ventilation. Among admission diagnoses, cardiac arrhythmia was linked to a lack of long-term MV requirement, whereas cerebral infarction and acute pancreatitis were associated with long-term MV requirement. Dependence on an aspirator or respirator, or a pre-existing diagnosis of chronic obstructive pulmonary disease, was a strong indicator of a high risk for long-term ventilation, while pulmonary or abdominal metastasis was associated with a lack of long-term MV requirement. In terms of operations and procedures in the 365 days prior to the ventilator case, tracheostomy was linked to long-term MV requirement, while the creation of a dialysis fistula, shunt, or bypass was associated with a lack of long-term MV requirement. During the ventilation case up to 95 h after intubation, procedures such as computed tomography and/or magnetic resonance imaging of the cranium with contrast medium, tracheostomy, chest tube positioning, treatment in a special bed, transfusion of plasma components and genetically engineered plasma proteins, extracorporeal lung support, and complex treatment for colonization or infection with multidrug-resistant pathogens were associated with long-term MV requirement. In contrast, radical cervical lymphadenectomy and autologous blood collection and transfusion were associated with lack of long-term MV requirementoutcome. Bronchoscopy or native computed tomography of the chest did not emerge as relevant factors in this analysis regarding subsequent long-term ventilation. However, these two variables were retained in the analysis based on the aforementioned methodology and were therefore continued to be included in the study. The prevalence (%) of the various risk factors and their association with long-term ventilation (OR (95% CI)) in the overall cohort and separately for women and men are shown in Table 2.

**Table 2:** Prevalence of various risk factors and their association with long-term ventilation in the overall cohort, as well as separately for women and men.

Predictor	All		Female		Male		Difference
	Prevalence [%]	OR (95% CI)	Prevalence [%]	OR (95% CI)	Prevalence [%]	OR (95% CI)	p-value
Baseline predictors							
Intercept							
Age [years]		1.00 (0.99–1.00)	-	0.99 (0.98–0.99)	-	1.00 (1.00–1.00)	
Sex [female]		0.91 (0.83–1.01)	-	-	-	-	
Nursing home accommodation	3.4	0.65 (0.49–0.87)	4.5	0.62 (0.91–2.16)	2.7	0.66 (0.46–0.94)	< 0.001
Diagnosis							
Pre-existing medical conditions (in 365 days prior to the MV case)							
Dementia	7.7	1.87 (1.25–2.78)	8.2	1.40 (0.91–2.16)	7.3	2.68 (1.44–5.01)	0.2228
Thyroiditis	1.2	0.88 (0.73–1.05)	2.3	1.05 (0.81–1.37)	0.4	0.84 (0.68–1.04)	< 0.001
Eating disorders	0.5	2.2 (1.2–4.03)	0.8	0.95 (0.46–1.98)	1.1	3.16 (1.37–7.26)	0.0015
Rheumatic mitral valve disease	1.5	2.37 (1.68–3.33)	2.3	2.74 (1.82–4.12)	0.6	1.71 (1.08–2.72)	< 0.001
Pneumothorax	0.6	2.24 (1.19–4.22)	0.5	1.76 (0.73–4.25)	0.9	1.94 (1.02–3.69)	0.8761
Peritonitis	1.0	0.56 (0.32–0.96)	1.2	0.70 (0.36–1.36)	0.9	0.55 (0.3–1)	0.1601
Admission diagnosis							
Cardiac arrhythmia	0.9	0.66 (0.39–1.12)	0.6	0.75 (0.29–1.93)	1.2	0.89 (0.54–1.46)	0.0012
Cerebral infarction	2.9	2.08 (1.58–2.73)	3.0	1.41 (0.97–2.07)	2.8	2.33 (1.72–3.14)	0.4503
Acute pancreatitis	0.6	3.57 (2.02–6.3)	0.3	4.73 (1.62–13.78)	0.7	3.01 (1.69–5.37)	0.0166
Diseases (previous diseases, admission diagnosis, MV case)							
Pulmonary or abdominal metastasis	2.5	0.63 (0.45–0.88)	2.4	0.67 (0.41–1.09)	2.6	0.71 (0.5–1)	0.6658
COPD	30.7	1.85 (1.68–2.05)	29.3	1.79 (1.54–2.08)	31.5	1.73 (1.55–1.93)	0.0118
Dependence on aspirator and/or ventilator (Non-invasive ventilation)	5.0	6.1 (4.8–7.76)	4.6	5.56 (3.97–7.77)	5.2	6.19 (4.81–7.96)	0.2251
Operations and procedures							
Operations and procedures in the 365 days prior to the MV case							
Tracheostomy. permanent or temporary	3.3	2.53 (1.96–3.28)	3.2	3.23 (2.24–4.66)	3.3	2.73 (2.06–3.61)	0.8068
Creation of a dialysis fistula. shunt or bypass	0.8	0.34 (0.17–0.65)	0.4	0.24 (0.05–1.13)	1.0	0.49 (0.27–0.9)	< 0.001
Operations and procedures during theMV case up to 95 h after intubation							
Bronchoscopy	35.1	1.15 (1.04–1.27)	33.4	1.20 (1.03–1.38)	36.1	1.15 (1.04–1.28)	0.0029
Native computed tomography of the chest	13.2	1.16 (1.01–1.33)	12.5	1.16 (0.95–1.43)	13.7	1.04 (0.9–1.21)	0.0695
Computed tomography and/or magnetic resonance imaging of the cranium with imaging contrast medium	10.7	1.34 (1.15–1.56)	10.8	1.22 (0.99–1.52)	10.7	1.31 (1.11–1.54)	0.832
Operations on the spinal cerebrospinal fluid system (drainage. shunt. catheter; also. removal)	0.4	2.54 (1.33–4.86)	0.4	5.69 (1.92–16.83)	0.5	2.01 (0.97–4.18)	0.7901
Tracheostomy, permanent or temporary	12.8	4.17 (3.63–4.78)	12.5	4.44 (3.63–5.41)	12.9	3.63 (3.12–4.22)	0.5367
Radical cervical lymphadenectomy	0.7	0.25 (0.13–0.5)	0.6	0.09 (0.02–0.39)	0.8	0.26 (0.13–0.51)	0.4943
Chest tube	10.8	1.35 (1.16–1.57)	11.0	1.27 (1.03–1.58)	10.6	1.23 (1.05–1.45)	0.5556
Positioning treatment in a special bed**	2.4	2.37 (1.78–3.15)	2.2	2.07 (1.35–3.17)	2.4	2.27 (1.66–3.13)	0.5035
Autologous blood collection and transfusion	2.7	0.71 (0.51–0.97)	2.1	0.6 (0.35–1.01)	3.0	0.83 (0.61–1.15)	0.0064
Transfusion of plasma components and genetically engineered plasma proteins	13.0	1.46 (1.27–1.69)	12.4	1.4 (1.14–1.73)	13.4	1.43 (1.22–1.66)	0.1440
PECLA. ECCO2R. vv- und va ECMO und Pre-ECMO therapy	3.4	1.43 (1.11–1.85)	2.6	0.93 (0.6–1.42)	3.9	1.62 (1.25–2.1)	< 0.001
Complex treatment for colonization or infection with multidrug-resistant pathogens	6.1	1.46 (1.2–1.78)	5.7	1.36 (1.03–1.81)	6.4	1.44 (1.17–1.77)	0.1166

The prevalence (%) of risk factors is shown in the first column for the overall cohort. The second column presents the independent associations with the risk of long-term ventilation (OR (95% CI)) according to the criteria outlined in Table S1. The subsequent columns show the associations from models created separately for men and women. Abreviations: COPD: Chronic Obstructive Pulmonary Disease, PECLA: Pumpless Extracorporeal Lung Assist ECCO2R: Extracorporeal Carbon Dioxide Removalvv-ECMO: Venovenous Extracorporeal Membrane Oxygenation va-ECMO: Venoarterial Extracorporeal Membrane Oxygenation*at least 3 completed months, **e.g. positioning in a rotating or.

### Sex differences in relation to the identified risk factors

**Fig. 2:**
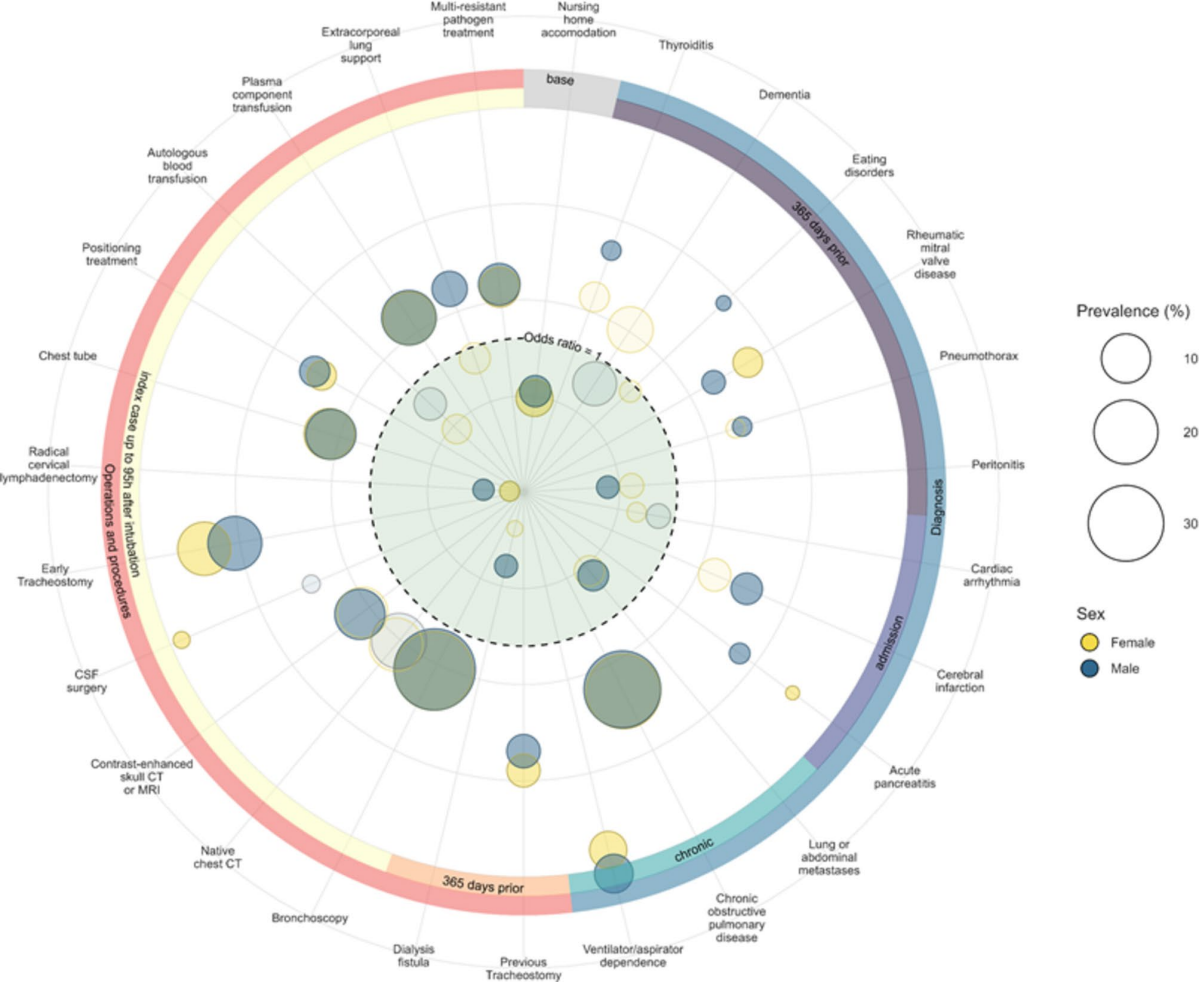
Odds ratios and prevalence of risk factors for long-term mechanical ventilation in men and women. The figure illustrates the odds ratios of individual risk factors along with their prevalence. Each circle represents a risk factor, with yellow circles corresponding to women and blue circles corresponding to men. Risk factors that were found to be non-significant in the individual analyses are shown as transparent circles. The size of each circle reflects the prevalence of the risk factor within the respective population. The outermost circle categorizes the different risk factors: blue represents diagnoses (chronic diseases at admission, admission diagnoses, and diagnoses within the last 365 days prior to admission), and red represents surgeries and procedures during the inpatient stay (hospital stay, 365 days prior to admission, and within the first 96 h of the inpatient stay). The area of each circle indicates the prevalence in the population. The dotted circle marks a hazard ratio of 1, with the distance of each comorbidity from the circle’s center reflecting the magnitude of the odds ratio (as shown in Table 2). Hazard ratios smaller than 1 are plotted inward, while those greater than 1 are plotted outward.

In regression analyses conducted separately for each sex, differences in the relevance of various risk factors were observed. Specifically, the pre-existing medical condition thyroiditis was associated with a higher risk for subsequent long-term ventilation in men. In contrast, rheumatic mitral valve disease was linked to a higher risk for long-term ventilation in women. Additionally, eating disorders and pneumothorax were identified as relevant risk factors exclusively in men. Among the admission diagnoses, cerebral infarction was associated with a higher risk for men, while acute pancreatitis was linked to a higher risk for women. Among the identified pre-existing conditions documented during the index case and operations and procedures in the 365 days prior to the ventilator case, no significant differences between the sexes were observed. However, regarding operations and procedures during the ventilation case up to 96 h after intubation, a trend was observed indicating a stronger unfavorable influence of early tracheotomy in women. Additionally, extracorporeal lung support was associated with an increased risk for subsequent long-term ventilation only in men. See Table 2 and Fig. 2: Odds Ratios and Prevalence of Risk Factors for Long-Term Mechanical Ventilation in Men and Women.

## Discussion

This longitudinal cohort study is the first comprehensive investigation into sex differences in risk factors for long-term ventilation within an at-risk population. An analysis of over 12,000 hospital admissions involving invasive mechanical ventilation for more than 96 h revealed that sex significantly affects both early intensive care and risk factors for subsequent long-term ventilation. Men were more likely to require invasive long-term ventilation (33.7% vs. 31.2%, p = 0.004) and had more hours of mechanical ventilation (400.0 ± 377.9 vs. 373.0 ± 341.8, p < 0.001). Hospital mortality rates were similar for both sexes. The increased likelihood of long-term ventilation in men was primarily due to a higher burden of risk factors. After adjusting for these factors, sex alone was not a significant determinant. Notably, women exhibited distinct healthcare utilization patterns and differences in interventions during the critical early care period, receiving fewer relevant therapeutic interventions. Additionally, biological differences played a crucial role, with women showing a unique pattern of comorbidities that significantly influenced the course of ventilation ^14^. Notably, these comorbidities not only differed in prevalence between the sexes, but also had different effects on the likelihood and outcome of long-term ventilation. In terms of baseline data, it was noted that women were more likely to be coming from nursing homes, which is likely related to the fact that men are often older than their wives, allowing women to care for their husbands. In contrast, the reverse scenario is less common, making women more likely to require nursing care themselves ^16^. An interesting finding was the lower frequency of certain therapeutic interventions in the first 96 h of ICU care, which were identified as relevant factors influencing subsequent long-term ventilation outcomes in the overall population. Women received fewer treatments, such as bronchoscopy, extracorporeal lung support, autologous blood transfusion. This difference may reflect varying treatment protocols or clinical decision-making factors, which could influence long-term ventilation outcomes. Whether this reflects a lower treatment need in women remains unclear. A study from Taiwan investigating sex-related differences in patients receiving ECMO found that despite advances in ECMO techniques, it remains underutilized in eligible female patients ^17^. A large, retrospective single-center analysis of adult ICU patients admitted to the University Hospital of Regensburg between January 2010 and December 2017 yielded similar results. In this study, the researchers analyzed a cohort of 26,711 ICU patients to investigate sex-related differences in treatment and mortality. After adjustment for severity of disease and outcome, ICU treatment differs between men and women. Men were more likely than women to undergo tracheostomy and ECMO. These findings align with the results of the current study, which also identified male sex as a significant factor related to the use of ECMO ^18^.The findings reveal sex-specific differences in the prevalence of selected comorbidities that influence the overall outcome parameters of long-term ventilation. Comorbidities such as thyroiditis, rheumatic valve disease were observed to occur significantly more frequently in women. These conditions were either documented within the 365 days prior to the index admission or recorded as pre-existing diagnoses or procedures upon admission. Importantly, only comorbidities with a measurable impact on the outcome were included in the current analysis, emphasizing their potential role in sex-based disparities in long-term ventilation outcomes. Interestingly, thyroiditis—though more common in women—appears to be associated with a higher risk of long-term mechanical ventilation in men This finding suggests a potential sex-specific effect of thyroiditis on mechanical ventilation outcomes. While more common in women, its impact appears greater in men, possibly due to differences in disease biology or management. Both hyper- and hypothyroidism may impair respiratory function via muscle weakness, increasing the risk of long-term ventilation. ^19,20^. Additionally, diagnostic bias may contribute to delayed recognition, particularly in men, resulting in more advanced disease at the time of detection and potentially amplifying its impact on respiratory outcomes. A similar pattern may apply to eating disorders (ED), which are generally more common in women. Men with EDs may be less likely to seek professional help, and when they do, their symptoms are often more severe and associated with a higher burden of comorbidities. These sex-specific differences could partly explain why certain conditions, such as thyroiditis or EDs, appear to be stronger risk factors for long-term mechanical ventilation in men, while their effect in women may be mitigated by earlier mortality or competing risks. . This sex disparity in diagnosis and treatment may further impact the clinical course and outcomes of ED in patients requiring long-term mechanical ventilation ^21^. Notably in our analyses, EDs represent an independent risk factor for long-term ventilation only in men. In contrast to previous studies ^14^, cardiac arrhythmia did not have a significant impact on ventilation outcomes, neither in the overall cohort nor in either sex. However, the admission diagnoses of cerebral infarction and acute pancreatitis consistently emerged as relevant risk factors for subsequent long-term ventilation across all analyses. Notably, the odds for cerebral infarction were higher in men than in women, while for pancreatitis, the odds were higher in women than in men. These findings suggest that both ischemic stroke and acute pancreatitis are significant predictors of long-term ventilation, but their influence varies by sex. For ischemic stroke, men appear to be at a higher risk, which contrasts with the broader stroke literature where stroke outcomes tend to be more severe for women, particularly regarding functional recovery. This difference may be partly due to women often not receiving the correct initial treatment and being older at stroke onset ^22^. However, in our study, the focus is not on post-stroke recovery but rather on the risk factors for requiring long-term ventilation. The biological and clinical factors influencing the need for ventilation in stroke patients may differ significantly from those affecting stroke severity or rehabilitation outcomes, which could explain these discrepancies. As mentioned earlier, men are more frequently treated with extracorporeal lung support. Interestingly, however, this is associated with a relevant risk of subsequent long-term ventilation only in men. We can only speculate as to why this is the case. A potential explanation for this could be related to sex-specific differences in the underlying pathophysiology and response to treatment. Men may experience more severe forms of respiratory failure or associated comorbidities, which could make them more reliant on extracorporeal lung support. Additionally, delays in diagnosis or treatment in men might lead to more advanced disease at the time of intervention, thereby increasing the risk of subsequent long-term ventilation. However, further research is necessary to explore these potential mechanisms.

## Limitations

The main limitation of our study lies in the use of healthcare service data designed for reimbursement, which brings associated challenges such as incomplete or non-specific coding and potential inaccuracies in clinical documentation. A potential selection bias also exists due to the fact that we analyzed data from a single health insurance provider, AOK, within a single federal state in Germany. While AOK is the largest health insurance fund in the country, its insured population may differ in structure from those of other health insurance providers, particularly private insurers, which could introduce distortion effects.

## Conclusion

Our analysis, the first of its kind in such a large population, highlights significant sex differences in the absolute numbers, prevalence, and impact of risk factors for long-term mechanical ventilation (MV). These findings underscore the importance of incorporating sex-specific considerations in the management of patients requiring prolonged ventilation, suggesting that personalized treatment strategies could be beneficial. Further investigation into these differences may contribute to the development of more individualized therapeutic approaches.

## Supplementary Information

Supplementary Information 1.

Supplementary Information 2.

Supplementary Information 3.

Supplementary Information 4.

## Data Availability

The datasets used and/or analyzed during the current study are available from the corresponding author on reasonable request.
