# Supplementary material for: Sex-specific differences in risk factors and outcomes for long-term mechanical ventilation: a longitudinal cohort analysis of claims data
Source: Sci Rep. 2025 Oct 8;15:35051. doi: 10.1038/s41598-025-22399-z (PMC12508137; doi:10.1038/s41598-025-22399-z)
Supplement: Supplementary file 1 — Supplementary Information 1. [file 41598_2025_22399_MOESM1_ESM.pptx]

## Slide 1
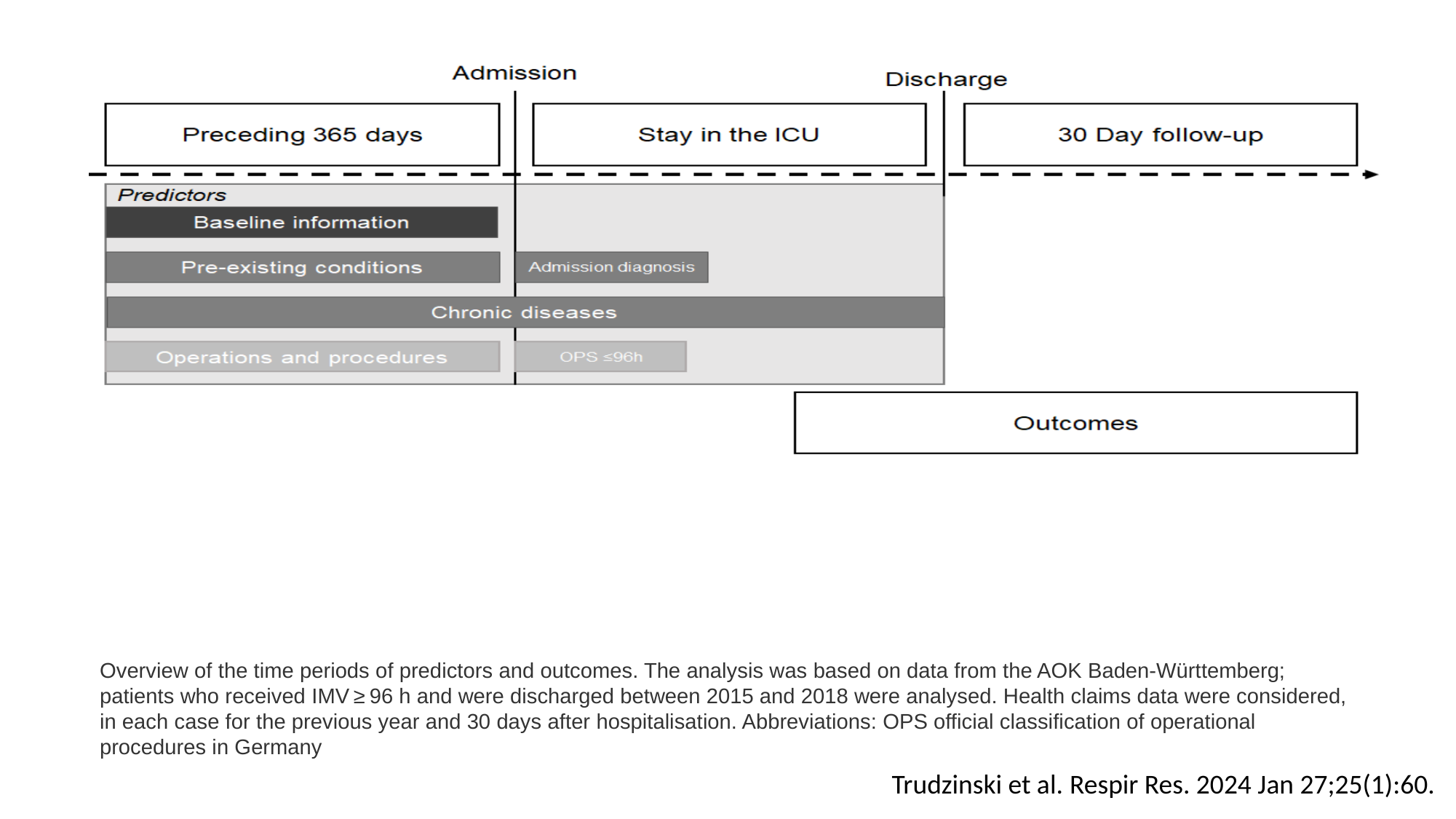

#
Overview of the time periods of predictors and outcomes. The analysis was based on data from the AOK Baden-Württemberg; patients who received IMV ≥ 96 h and were discharged between 2015 and 2018 were analysed. Health claims data were considered, in each case for the previous year and 30 days after hospitalisation. Abbreviations: OPS official classification of operational procedures in Germany
Trudzinski et al. Respir Res. 2024 Jan 27;25(1):60.
