# Supplementary material for: Sex-specific differences in risk factors and outcomes for long-term mechanical ventilation: a longitudinal cohort analysis of claims data
Source: Sci Rep. 2025 Oct 8;15:35051. doi: 10.1038/s41598-025-22399-z (PMC12508137; doi:10.1038/s41598-025-22399-z)
Supplement: Supplementary file 2 — Supplementary Information 2. [file 41598_2025_22399_MOESM2_ESM.docx]

| **Criteria** | **Definition** |
| --- | --- |
| Evidence of home invasive ventilation after discharge | |
| Initiation of home mechanical ventilation  - Invasive HMV after weaning failure and within 30 days. | OPS 8716.01 |
| Control or optimisation of a previously initiated HMV within 30 days | OPS 8716.11 |
| Termination of previously initiated home ventilation within 30 days | OPS 8716.21 |
| Tracheostomy ventilator aids prescribed after start of ventilation and within 30 days of discharge | Nr. 12.50.99.0002* |
| Inpatient: dependence (long term) on respirator after start of ventilation AND  Care of a tracheostoma after the start of ventilation and within 30 days after discharge | ICD Z99.1 |
|  | ICD Z43.0 |
| Outpatient: Dependence (long-term) on respirator  AND.  Care of a tracheostoma in the quarter following the end of the respirator claim. | ICD Z99.1 |
|  | ICD Z43.0 |
| Total duration of ventilation ≥ 500 hours | |
| Total duration of ventilation is 500 or more hours | Ventilation hours ≥ 500 |
| Re-hospitalisation with (re)prolonged ventilation | |
| Re-hospitalisation with (re)prolonged ventilation within 30 days after discharge | Re-hospitalisation with initiation of prolonged ventilation within 30 days of discharge (with ventilation hours ≥ 96). |

**Table S1: Definition of Outcomes**

The table shows the different definitions of the three outcomes studied; long-term IMV defined as evidence of invasive mechanical home ventilation, IMV ≥ 500h and/or readmission with (re)prolonged ventilation. Abbreviations: HMV invasive mechanical home ventilation, IMV invasive mechanical ventilation, ICD international statistical classification of diseases and related health problems, OPS official classification of operational procedures in Germany. * the specified no. 12.50.99.0002 is an AOK-BW specific code.
