# Supplementary material for: Sex-specific differences in risk factors and outcomes for long-term mechanical ventilation: a longitudinal cohort analysis of claims data
Source: Sci Rep. 2025 Oct 8;15:35051. doi: 10.1038/s41598-025-22399-z (PMC12508137; doi:10.1038/s41598-025-22399-z)
Supplement: Supplementary file 3 — Supplementary Information 3. [file 41598_2025_22399_MOESM3_ESM.docx]

| Baseline predictors |  |
| --- | --- |
| *Diagnosis* |  |
| *Pre-existing medical conditions (in 365 days prior to the ventilator case).* |  |
| Dementia | ICD F00-F03 |
| Thyroiditis | ICD E06 |
| Eating disorders | ICD F50 |
| Rheumatic mitral valve disease | ICD I05 |
| Pneumothorax | ICD J93 |
| Peritonitis | ICD K65 |
| Admission diagnosis |  |
| Cardiac arrhythmia | ICD I49 |
| Cerebral infarction | ICD I63 |
| Acute pancreatitis | ICD K85 |
| *Diseases (previous disease. admission diagnosis. ventilation case).* |  |
| Pulmonary or abdominal metastasis | ICD C78 |
| COPD | ICD J44 |
| Dependence on aspirator and/or respirator*. | ICD Z99.0. Z99.1 |
| Operations and procedures |  |
| *Operations and procedures in the 365 days prior to the ventilator case.* |  |
| Tracheostomy. permanent or temporary | OPS 5311. 5312 |
| Creation of a dialysis fistula. shunt or bypass | OPS 5393 |
| *Operations and procedures during the ventilation case up to 95h after intubatio* |  |
| Bronchoscopy | OPS 1620 |
| Native computed tomography of the chest | OPS 3202 |
| Computed tomography and/or magnetic resonance imaging of the the cranium with imaging contrast medium | OPS 3220. 3820 |
| Operations on the spinal cerebrospinal fluid system (drainage. shunt. catheter; also. removal) | OPS 5038 |
| Tracheostomy, permanent or temporary | OPS 5311. 5312 |
| Radical cervical lymphadenectomy | OPS 5403 |
| Chest tube | OPS 8144 |
| Positioning treatment in a special bed** | OPS 8390.0 |
| Autologous blood collection and transfusion | OPS 8803 |
| Transfusion of plasma components and genetically engineered plasma proteins | OPS 8810 |
| PECLA. ECCO2R. vv- und va ECMO und Pre-ECMO therapy | OPS 8852 |
| Complex treatment for colonization or infection with multidrug-resistant pathogens | OPS 8987 |

**Supplementary Table 2: ICD and OPS codes of the diagnoses and procedures used in the final model**
