# Supplementary material for: Sex-specific differences in risk factors and outcomes for long-term mechanical ventilation: a longitudinal cohort analysis of claims data
Source: Sci Rep. 2025 Oct 8;15:35051. doi: 10.1038/s41598-025-22399-z (PMC12508137; doi:10.1038/s41598-025-22399-z)
Supplement: Supplementary file 4 — Supplementary Information 4. [file 41598_2025_22399_MOESM4_ESM.doc]

STROBE Statement—Checklist of items that should be included in reports of ***cohort studies***

|  | Item No | Recommendation |
| --- | --- | --- |
| **Title and abstract** | 1 | (*a*) Indicate the study’s design with a commonly used term in the title or the abstract  Title p1 |
|  |  | (*b*) Provide in the abstract an informative and balanced summary of what was done and what was found  Line 66-92 |
| Introduction | | |
| Background/rationale | 2 | Explain the scientific background and rationale for the investigation being reported  Line 88-94 |
| Objectives | 3 | State specific objectives, including any prespecified hypotheses  Line 95-147 |
| Methods | | |
| Study design | 4 | Present key elements of study design early in the paper  Line 88-94 |
| Setting | 5 | Describe the setting, locations, and relevant dates, including periods of recruitment, exposure, follow-up, and data collection  Line 95-147 |
| Participants | 6 | (*a*) Give the eligibility criteria, and the sources and methods of selection of participants. Describe methods of follow-up  Line 137-147 |
|  |  | (*b*) For matched studies, give matching criteria and number of exposed and unexposed  *N/A* |
| Variables | 7 | Clearly define all outcomes, exposures, predictors, potential confounders, and effect modifiers. Give diagnostic criteria, if applicable  Line 198-210 |
| Data sources/ measurement | 8* | For each variable of interest, give sources of data and details of methods of assessment (measurement). Describe comparability of assessment methods if there is more than one group  Line 161-195 |
| Bias | 9 | Describe any efforts to address potential sources of bias  119-135 |
| Study size | 10 | Explain how the study size was arrived at  See Figure 1 |
| Quantitative variables | 11 | Explain how quantitative variables were handled in the analyses. If applicable, describe which groupings were chosen and why  119-135 |
| Statistical methods | 12 | (*a*) Describe all statistical methods, including those used to control for confounding  119-135 |
|  |  | (*b*) Describe any methods used to examine subgroups and interactions  119-135 |
|  |  | (*c*) Explain how missing data were addressed  Complete data set for the analysed parameters |
|  |  | (*d*) If applicable, explain how loss to follow-up was addressed  N/A |
|  |  | (*e*) Describe any sensitivity analyses  N/A |
| Results | | |
| Participants | 13* | (a) Report numbers of individuals at each stage of study—eg numbers potentially eligible, examined for eligibility, confirmed eligible, included in the study, completing follow-up, and analysed  Figure 1 |
|  |  | (b) Give reasons for non-participation at each stage  N/A |
|  |  | (c) Consider use of a flow diagram  Figure 1 |
| Descriptive data | 14* | (a) Give characteristics of study participants (eg demographic, clinical, social) and information on exposures and potential confounders  Table 1 |
|  |  | (b) Indicate number of participants with missing data for each variable of interest  none |
|  |  | (c) Summarise follow-up time (eg, average and total amount)  137-140 |
| Outcome data | 15* | Report numbers of outcome events or summary measures over time  Table 1 |
| Main results | 16 | (*a*) Give unadjusted estimates and, if applicable, confounder-adjusted estimates and their precision (eg, 95% confidence interval). Make clear which confounders were adjusted for and why they were included  Table 2 |
|  |  | (*b*) Report category boundaries when continuous variables were categorized  Supplement |
|  |  | (*c*) If relevant, consider translating estimates of relative risk into absolute risk for a meaningful time period  N/A |
| Other analyses | 17 | Report other analyses done—eg analyses of subgroups and interactions, and sensitivity analyses  none |
| Discussion | | |
| Key results | 18 | Summarise key results with reference to study objectives  210-232 |
| Limitations | 19 | Discuss limitations of the study, taking into account sources of potential bias or imprecision. Discuss both direction and magnitude of any potential bias  293-299 |
| Interpretation | 20 | Give a cautious overall interpretation of results considering objectives, limitations, multiplicity of analyses, results from similar studies, and other relevant evidence  300-306 |
| Generalisability | 21 | Discuss the generalisability (external validity) of the study results  293-299 |
| Other information | | |
| Funding | 22 | Give the source of funding and the role of the funders for the present study and, if applicable, for the original study on which the present article is based  334-335 |

*Give information separately for exposed and unexposed groups.

**Note:** An Explanation and Elaboration article discusses each checklist item and gives methodological background and published examples of transparent reporting. The STROBE checklist is best used in conjunction with this article (freely available on the Web sites of PLoS Medicine at http://www.plosmedicine.org/, Annals of Internal Medicine at http://www.annals.org/, and Epidemiology at http://www.epidem.com/). Information on the STROBE Initiative is available at http://www.strobe-statement.org.
